# Personalized CT-based radiomics nomogram preoperative predicting Ki-67 expression in gastrointestinal stromal tumors: a multicenter development and validation cohort

**DOI:** 10.1186/s40169-020-0263-4

**Published:** 2020-01-31

**Authors:** Qing-Wei Zhang, Yun-Jie Gao, Ran-Ying Zhang, Xiao-Xuan Zhou, Shuang-Li Chen, Yan Zhang, Qiang Liu, Jian-Rong Xu, Zhi-Zheng Ge

**Affiliations:** 1 Division of Gastroenterology and Hepatology, Key Laboratory of Gastroenterology and Hepatology, Ministry of Health, Renji Hospital, School of Medicine, Shanghai Jiao Tong University, Shanghai Institute of Digestive Disease, Shanghai, China; 2 Department of Radiology, Zhongshan Hospital, Fudan University and Shanghai Institute of Medical Imaging, Shanghai, China; 3 Department of Radiology, Sir Run Run Shaw Hospital (SRRSH) of School of Medicine, Zhejiang University, Hangzhou, China; 4 Department of Radiology, First Affiliated Hospital of Wenzhou Medical University, Wenzhou, 325000 China; 5 Department of Pathology, Renji Hospital, School of Medicine, Shanghai Jiao Tong University, 160 Pujian Road, Shanghai, 200025 China; 6 Department of Radiology, Renji Hospital, School of Medicine, Shanghai Jiao Tong University, No. 1630, Dongfang Road, Pudong, Shanghai, 200120 China

**Keywords:** Radiomic signature, Gastrointestinal stromal tumor, Ki-67, Prediction

## Abstract

**Background and Aim:**

To develop and validate radiomic prediction models using contrast-enhanced computed tomography (CE-CT) to preoperatively predict Ki-67 expression in gastrointestinal stromal tumors (GISTs).

**Method:**

A total of 339 GIST patients from four centers were categorized into the training, internal validation, and external validation cohort. By filtering unstable features, minimum redundancy, maximum relevance, Least Absolute Shrinkage and Selection Operator (LASSO) algorithm, a radiomic signature was built to predict the malignant potential of GISTs. Individual nomograms of Ki-67 expression incorporating the radiomic signature or clinical factors were developed using the multivariate logistic model and evaluated regarding its calibration, discrimination, and clinical usefulness.

**Results:**

The radiomic signature, consisting of 6 radiomic features had AUC of 0.787 [95% confidence interval (CI) 0.632–0.801], 0.765 (95% CI 0.683–0.847), and 0.754 (95% CI 0.666–0.842) in the prediction of high Ki-67 expression in the training, internal validation and external validation cohort, respectively. The radiomic nomogram including the radiomic signature and tumor size demonstrated significant calibration, and discrimination with AUC of 0.801 (95% CI 0.726–0.876), 0.828 (95% CI 0.681–0.974), and 0.784 (95% CI 0.701–0.868) in the training, internal validation and external validation cohort respectively. Based on the Decision curve analysis, the radiomics nomogram was found to be clinically significant and useful.

**Conclusions:**

The radiomic signature from CE-CT was significantly associated with Ki-67 expression in GISTs. A nomogram consisted of radiomic signature, and tumor size had maximum accuracy in the prediction of Ki-67 expression in GISTs. Results from our study provide vital insight to make important preoperative clinical decisions.

## Introduction

Gastrointestinal stromal tumors (GISTs) are the most commonly diagnosed subepithelial tumors in the gastrointestinal tract, histologically heterogeneous, biologically diverse, and challenging to predict their malignant potential [1, 2].

Recently, a number of risk classification systems have been developed to predict biological behaviors, including National Institutes of Health (NIH) modified criteria [2], National Comprehensive Cancer Network (NCCN) criteria [3], and the Armed Forces Institute of Pathology (AFIP) criteria [4]. Although NIH modified criteria and AFIP criteria are the most commonly used tools to assess the risk of malignant potential in GISTs, a significantly risk is associated with poor survival chances in patients with GISTs [5]. Also, the clinical behaviors and outcomes are very dynamic, especially among GISTs, and fall under the high-risk category.

Ki-67 nucleoprotein, a key marker associated with cell growth and tumor heterogeneity, has increased expression from the G1 phase to mitosis with a sudden decline in the in the expression level in the G0 phase [6]. Instead of reflecting only during the M phase of the cell cycle. As measured by the mitotic count, Ki-67 is highly expressed in most of the proliferating cells except in G0 cells and is considered as a universal risk factor of malignancy in GISTs [7]. Multiple studies have demonstrated the association of Ki-67 higher expression with larger tumor size, higher mitotic rate, higher risk of malignancy and poor disease prognosis [7–12]. Previous studies have shown that high Ki-67 expression was an independent risk factor for high malignant potential and could help classify GISTs with a combination of mitotic rate and Ki-67 expression [10–12]. Also, independent of the NIH classification, the Ki-67 index has shown its prediction efficacy in the prediction of survival of patients with GISTs after treatment, including disease-specific survival and recurrence [7, 8, 10]. More importantly, it was found that Ki-67 expression was significantly associated with KIT mutation or PDGFRA mutation [13, 14]. Patients with PDGFRA mutation responded more poorly to the adjuvant imatinib and may not be considered for adjuvant imatinib. Therefore, Ki67 expression can also be used as a reference tool to predict whether patients are suitable for adjuvant imatinib administration or not.

The conventional method to detect the expression level of Ki-67 utilizes samples from either pre-operative fine-needle aspiration biopsy [15, 16] or from surgical procedures. Regardless, due to relatively smaller sample size and heterogeneity in the tumor samples, Ki-67 expression assessment based on invasive biopsy may not be a true representative of entire GISTs [15, 16] and limites its use in preoperative assessment of GISTs. Also, immunohistochemistry examination of Ki-67 expression in the surgical specimen for patients receiving presurgical adjuvant imatinib therapy may be underestimated and not accurate [15, 16]. Therefore, an accurate and a noninvasive tool is required to assess the preoperative Ki-67 expression status in patients with GISTs more accurately and comprehensively. In addition, a new tool will potentially help physicians not only in making right decision on the adjuvant imatinib administration, but also in developing a follow up post-operative plan for patients with large and unresected GISTs. In this paper we have developed a Radiomics prediction model, a novel tool that extracts hundreds of quantitative features based on shape, intensity, size or volume of the target lesions. Recently Radiomics prediction model has gained attention in the diagnosis of cancers [17, 18]. Previous studies have shown high accuracy of radiomics in the assessment of biological behavior of GISTs comprehensively, including malignant potential [19, 20], mitotic rate [19], recurrence [21]. However, to the best of our knowledge, this is the first ever study that investigates whether radiomics can be used as a tool to assess Ki-67 expression status in GISTs. In this study, we aimed to develop and validate a radiomic signature and radiomics based nomogram to predict the Ki-67 expression label in patients with GISTs.

## Patients and methods

### Patients

With the ethical approval obtained from all 4 participating hospitals, a total of 339 patients with GIST were enrolled in this retrospective study. Patients fulfilling the following inclusion criteria were included for analysis as follows: (1) patients who underwent surgery or endoscopic resection; (2) standard contrast-enhanced computed tomography (CE-CT) was performed within 15 days before the treatment; (3) GISTs were diagnosed with histology and immunohistochemistry examinations; (4) patients with possession of previously analyzed clinical and pathological variables. The exclusion criteria included patients, with a history of imatinib administration before surgery or with multiple detected GISTs.

Participants were divided into three independent cohorts: the training cohort, the internal validation cohort and external validation cohort. One hundred eighty-nine patients from the Renji hospital diagnosed between January 2011 and December 2018 were randomly assigned to either the training cohort (148 patients) and internal validation cohort (41 patients) in a 8:2 ratio. External validation cohort consisted of the remaining 150 patients diagnosed with GIST from the other three hospitals (Zhongshan Hospital, Sir Run Run Shaw Hospital and First Affiliated Hospital of Wenzhou Medical University) between January 2017 and December 2018.

### CT examination

The detailed information of the CT protocol was available in Additional file 1: A1 and Table S1.

### Immunohistochemistry

The Ki67 index was evaluated by immunohistochemistry within 7 days after surgical monoclonal mouse antihuman Ki-67 antibody was used to detect Ki-67 according to the manufacturer’s protocol. According to 50-fold microscopy, in each section, 1000 cells were randomly selected, and the Ki-67 positive cells were counted. According to the previous studies, the cut-off value for determining high Ki-67 expression was considered as ≥ 10% of positive cells [10–12, 22]. GISTs were classified into two groups: high Ki-67 expression (Ki-67 ≥ 10%) and low Ki-67 expression (Ki-67 < 10%).

### Clinical variable, and the primary outcome

Clinical and pathological data were collected, including age, gender, tumor location, tumor size and Ki67 index. Tumor size was recorded as clinical tumor size measured by the largest diameter on axial CT. The primary endpoint in the study was efficacy in prediction of high Ki-67 expression.

### Radiomic signature building

**Fig. 1:**
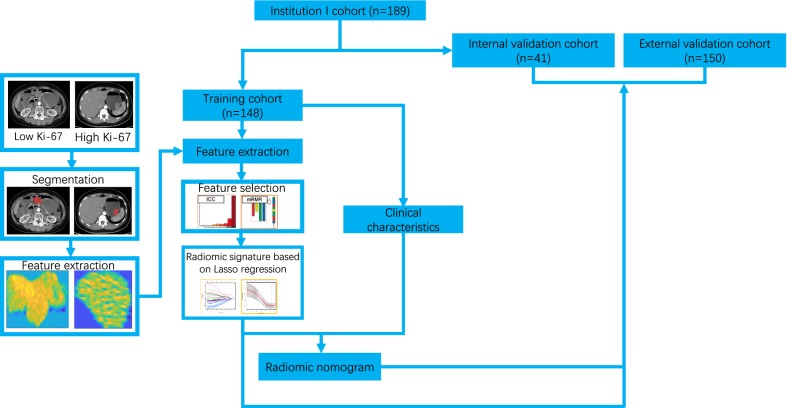
Schematic showing the workflow of the study. Based on the Ki67 expression profile, the tumor area were segmented and features were extracted. GIST patients were categorized into three different groups (training, internal validation, and external validation cohort), and the data from the training cohort were subjected to further downstream processing and clinical characterizations

All non-contrasted CT images were collected from Picture Archiving and Communication System for each GIST and then exported to the ITK-SNAP software (version 2.2.0; http://www.itksnap.org) for manual segmentation of the region of interest (ROI). For each patient, all slices of non-contrasted CT images were reviewed and the slice with the largest tumor area was selected. After that, 2D ROI for non-contrasted CT of the tumor was then delineated on the selected slice for each patient. Figure 1 shows the workflow of our study.

Radiomic features were extracted from the ROI of each GIST, including features of first order statistics, features of shape, features of grey-level co-occurrence matrix (GLCM), features of grey-level run-length matrix (GLRLM), features of grey-level size-zone matrix (GLSZM), features of gray-level dependence matrix (GLDM) using PyRadiomics on Python (version 3.7) [23]. The details of radiomic features extraction were described in Additional file 1: A2. As outlined in Additional file 1: A3, radiomic features selection and radiomic signature building were performed as follows: (1) feature reproducibility assessment using intra-/inter-class correlation coefficients (ICCs) was established [24]; (2) reservation of top features using minimum redundancy maximum relevance (mRMR) were ploted [25]; (3) signature building with least absolute shrinkage and selection operator (LASSO) algorithm was completed [26]. By following these three steps, the radiomic signature based on radiomic features of non-contrasted CT were built as predictors of high-malignant potential GISTs.

### Development of the radiomic nomogram

A multivariate logistic model was utilized to develop the radiomic nomogram. While developing the radiomic signature in the training cohort, potential risk factors for high Ki-67 expression, including age, tumor location, and tumor size were included. The backward step-wise selection was applied by using the likelihood ratio test with Akaike’s information criterion as the stopping rule [27]. To provide an easy and personalized quantitative tool to predict the probability of high Ki-67 expression, we built the radiomic nomogram based on the variable that were statistically significant in the multivariate logistic model.

### Statistical analysis

Statistical analysis was performed with R software (version 3.5.0) and Python (version 3.7) with P value less than 0.05 considered as statistical significance.

For development of a prediction model based on the logistic regression, a minimum of 10 events per predictor variable (EPV) is required for development [28]. In this study, five potential risk factors for high Ki-67 expression, including age, tumor location, tumor size, and our developed radiomic signature, were included for the development of radiomic nomogram. Therefore, at least 40 patients with high Ki-67 expression were required for the development cohort. About 25% of GISTs were diagnosed as GISTs with high Ki-67 expression and totally 160 patients were required to be included for the development cohort.

Predictive accuracy of the radiomic signature and radiomic nomogram was evaluated by the receiver operating characteristic (ROC) curve, and the area under ROC curve (AUC) was calculated in the same way as previously reported [29, 30]. To correct overfitting bias, a corrected AUC was calculated using bootstrapping validation (1000 bootstrap resamples) in the training cohort. Calibration of the radiomic nomogram was assessed by the calibration curve with Hosmer–Lemeshow test [27]. In addition, decision curve analysis was performed to evaluate clinical usefulness of the radiomic signature and radiomic nomogram by quantifying the net benefit at different threshold probabilities. An optimal cutoff value with the largest Youden index for classifying the patients into low- and high-risk groups based on the risk of high Ki67 expression was calculated. During the calculation, we used data from the training cohort, and applied to the internal validation, and external validation cohorts [31]. Accuracy, sensitivity, specificity, negative predictive value (NPV), and positive predictive value (PPV) were also calculated for the training cohort, internal validation and external validation cohorts using the respective defined optimal cutoff values.

## Results

### Patient characteristics

According to the Ki67 expression level, patients with GISTs were divided into two groups: GISTs with high Ki67 expression and GISTs with low Ki67 expression. As shown in the Table 1, we observed a positive correlation between the tumor size, and Ki67 expression. The tumor size was significantly associated with expression level of Ki67 in the univariate analysis model. In addition, only tumor location was significantly associated with expression level of Ki67 in the external validation data.

**Table 1:** Clinical characteristics of patients in the training cohort and validation cohort

	Training cohort			Internal validation cohort			External validation cohort		
	Low expression	High expression	P^*^	Low expression	High expression	P^*^	Low expression	High expression	P^*^
	N = 100	N = 48		N = 29	N = 12		N = 116	N = 34	
Age, mean ± SD, years	62.2 ± 12.66	63.08 ± 12.03	0.683	63 ± 13.1	60.17 ± 12.76	0.53	61.81 ± 10.30	59.91 ± 9.99	0.649
Sex			1.000			0.629			0.960
Female	47 (47.00%)	22 (45.83%)		14 (48.28%)	4 (33.33%)		71 (61.21%)	20 (58.82%)	
Male	53 (53.00%)	26 (54.17%)		15 (51.72%)	8 (66.67%)		45 (38.79%)	14 (41.18%)	
Location			0.662			0.184			0.007
Intestine	37 (37.00%)	21 (43.75%)		10 (34.48%)	7 (58.33%)		20 (17.24%)	14 (41.18%)	
Stomach	63 (63.00%)	27 (56.25%)		19 (65.52%)	5 (41.67%)		96 (82.76%)	20 (58.82%)	
Size (cm)			< 0.001			< 0.001			< 0.001
≤ 2	13 (13.00%)	1 (2.08%)		4 (13.79%)	0 (0%)		21 (18.1%)	1 (2.94%)	
2–5	54 (54.00%)	13 (27.08%)		14 (48.28%)	1 (8.33%)		68 (58.62%)	11 (32.35%)	
5–10	28 (28.00%)	19 (39.58%)		8 (27.59%)	4 (33.33%)		27 (23.28%)	15 (44.12%)	
> 10	5 (5.00%)	15 (31.25%)		3 (10.34%)	7 (58.33%)		0 (0)	7 (20.59%)	

* P value is calculated for difference of clinical characteristics between patients with low expression of Ki67 and patients with high expression of Ki67 by using the univariate analysis

### Feature selection and radiomic signature building

**Fig. 2:**
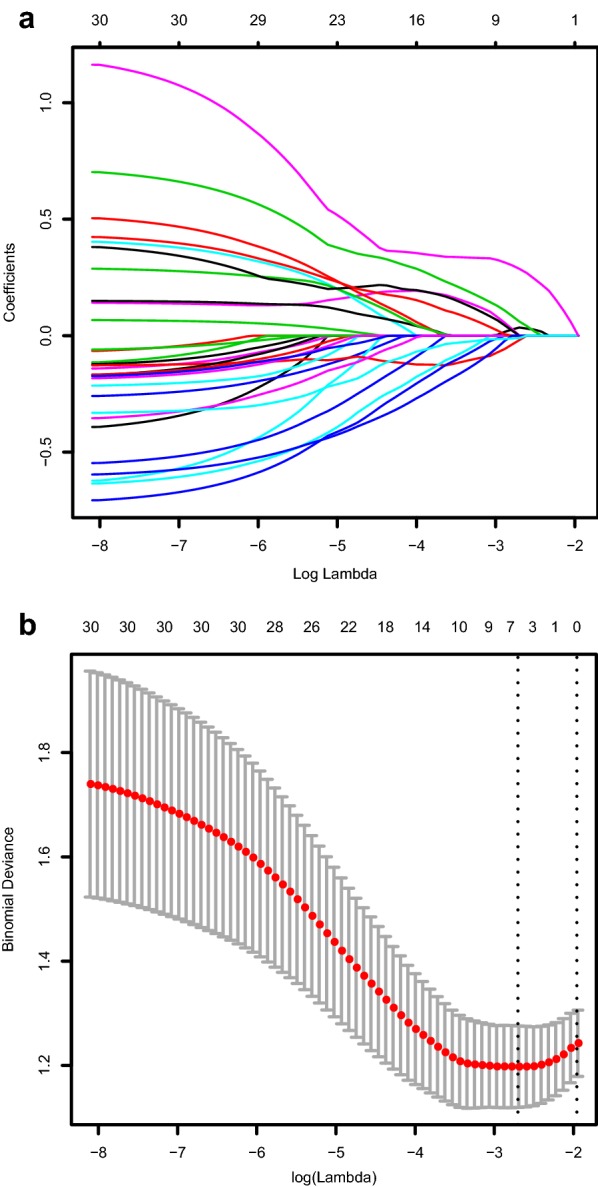
Texture feature selection using the least absolute shrinkage and selection operator (LASSO) binary logistic regression model. **a** LASSO coefficient profiles, displaying thirty texture features. A coefficient profile plot was produced against the log (lambda) sequence. Each colored line represents the coefficient of individual feature. **b** Tuning parameter (log lambda) selection in the LASSO model used tenfold cross-validation via minimum criteria. Vertical dotted lines were drawn at the selected λ values

**Fig. 3:**
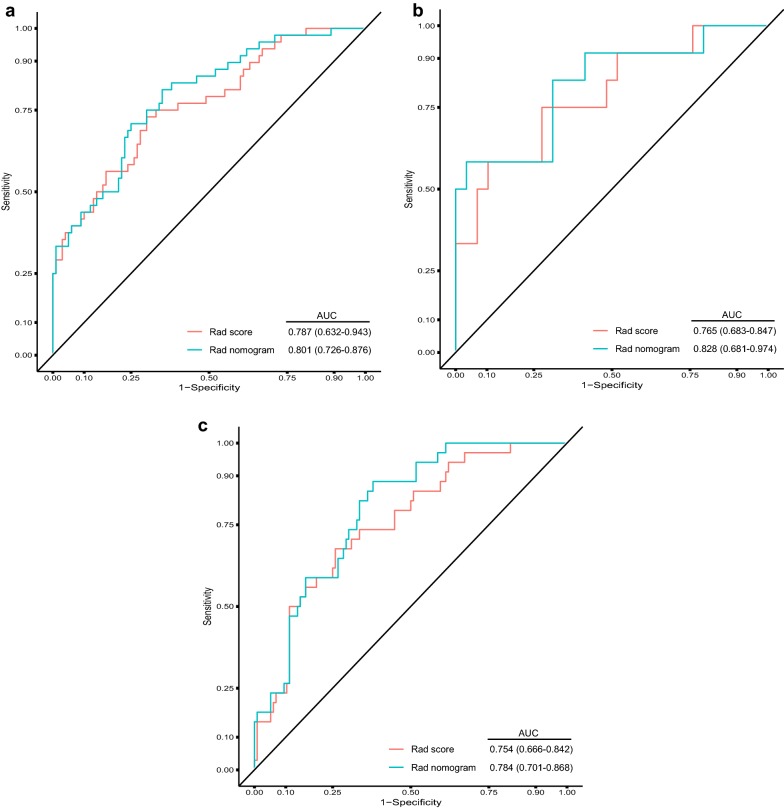
AUC of the Rad score and the Rad nomogram in prediction of high Ki67 expression presence in the development cohort (**a**), internal validation cohort (**b**), and external validation cohort (**c**), respectively. AUC: areas under the receiver operating characteristic curve

Fourty patients in the training cohort were randomly selected for segmentation by radiologist and re-segmented by the same radiologist 2 weeks after the initial segmentation to calculate the intra-/inter-class correlation coefficients for each extracted radiomic feature. These cases were then segmented by another radiologist for calculating of the intra-/inter-class correlation coefficients of each extracted radiomic feature. Using ICC of 0.80 (both intra-and inter ICC) as a cut-off for determining good reproducibility, a total of 423 radiomic features with good reproducibility were selected for next assessment. This was followed by calculating the mutual information (MI) for each included radiomic features. Radiomic features were ranked using the minimum redundancy maximum relevance (mRMR) algorithm and the only top 30 highest-ranking features were considered in our study. After application of LASSO logistic algorithm (Fig. 2), six out of the 30 radiomic features were finally used to develop the radiomic signature (Additional file 1: A4). As shown in the Fig. 3, AUC of the radiomic signature, developed by our group were found to be 0.787 [95% confidence interval (CI): 0.632–0.943], 0.765 (95% CI 0.683–0.847), 0.754 (95% CI 0.666–0.842) in the training cohort, internal validation cohort and external validation cohort, respectively.

### Development and validation of the radiomic nomogram

**Fig. 4:**
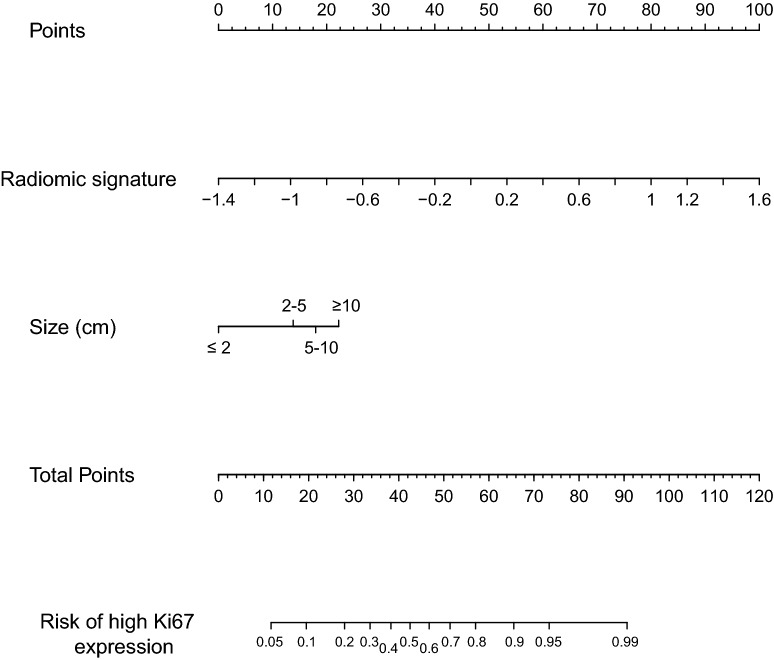
Development of the Radiomic nomogram. The range, radiomic signature, size, and Ki67 expression profiles were used to build the Radiomic nomogram. The probability of individual predictor were converted into scores according to the first scale Points. To use the nomogram, find the position of each variable on the corresponding axes, and a line was drawn to the points axis for the number of points, add the points from all of the variables, and draw a line from the total points axis to determine probability of high Ki67 expression at the lower line of the nomogram

In this study, the multivariate logistic model identified the tumor size and our developed radiomic signature as independent risk factors for high Ki67 expression (Table 2). These two factors were included to develop the radiomic nomogram, which was shown in the Fig. 4.

**Table 2:** Variable and coefficients of the radiomic nomogram

Variable	β	OR (95% CI)	*P*
Intercept	0.307	–	–
Radiomic signature (per 0.1 increase)	0.308	1.36 (1.1–1.68)	0.004
Size			
≤ 2	0	Reference	
2–5	1.304	3.68 (0.41–32.76)	0.242
5–10	1.631	5.11 (0.58–45.32)	0.143
> 10	2.442	11.5 (1.03–128.73)	0.048

*OR* odds ratio, *CI* confidence interval

**Fig. 5:**
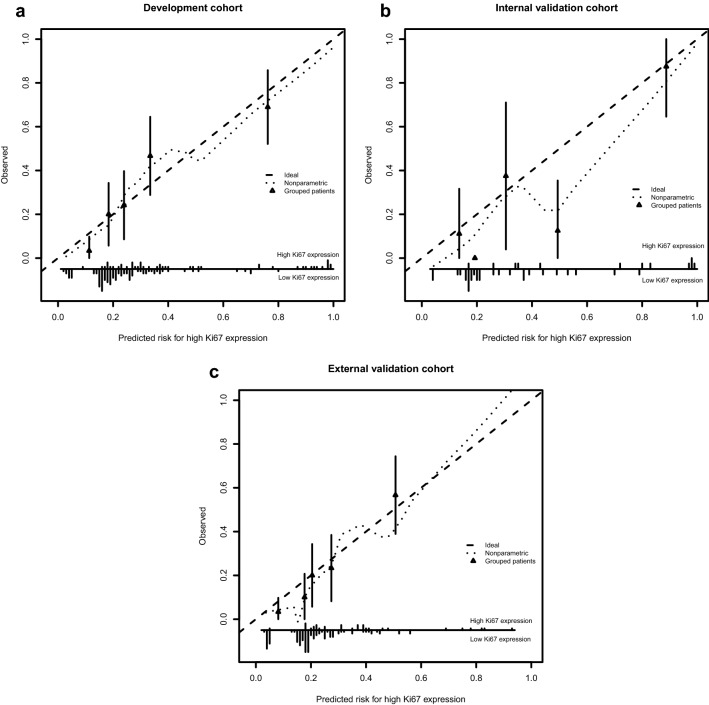
Validity of the predictive performance of the nomogram in estimating the risk of high Ki67 expression presence in the training cohort (**a**, n = 148), internal validation cohort (**b**, n = 41) and external cohort (**c**, n = 148). The distribution of the predicted probabilities value of high Ki67 expression presence is shown at the bottom of the graphs, separating those with high Ki67 expression (+) and low Ki67 expression (−). The triangles indicate the observed frequencies of high Ki67 expression presence by the deciles of the predicted probability

AUC (Fig. 3a) of the radiomic nomogram were 0.801 (95% CI 0.726–0.876) in the training cohort. We found a decent calibration curve, which demonstrated a good agreement between prediction and observation in the training cohort (Fig. 5a). In addition, the Hosmer–Lemeshow test demonstrated no statistical significance in the training cohort (P = 0.464). Results indicated no departure from perfect fit.

The nomogram that was developed in this study was validated both in the internal validation cohort and external validation cohort. AUC of the radiomic nomogram were recorded as 0.828 (0.681–0.974), 0.784 (0.701–0.868) in internal validation cohort (Fig. 3b) and external validation cohort (Fig. 3c), respectively. The Hosmer–Lemeshow test showed no significance in the training cohort (P = 0.464, Fig. 5a), the internal validation cohort (P = 0.444, Fig. 5b) and the external cohort (P = 0.215, Fig. 5c).

Based on the risk of high Ki67 expression for each patient according to our developed nomogram, a cut-off value of 0.27 was used to classify patients into high Ki67 expression group, and low Ki67 expression group. Based on the cut-off value, the nomogram was able to evaluate Ki67 expression status in about 70% patients, accurately (Table 3). In the validation cohort, PPV was more than 85% for prediction of high Ki67 expression.

**Table 3:** Diagnostic efficacy of the developed radiomic nomogram in assessment of high Ki67 expression of gastrointestinal stromal tumors

	Development cohort (%)	Internal validation cohort (%)	External validation cohort (%)
Accuracy	75.00	68.29	73.33
Sensitivity	75.00	83.33	58.82
Specificity	75.00	62.07	77.59
PPV	59.02	90.00	86.54
NPV	86.21	52.38	56.52

*PPV* positive predictive value, *NPV* negative predictive value

### Clinical significance

**Fig. 6:**
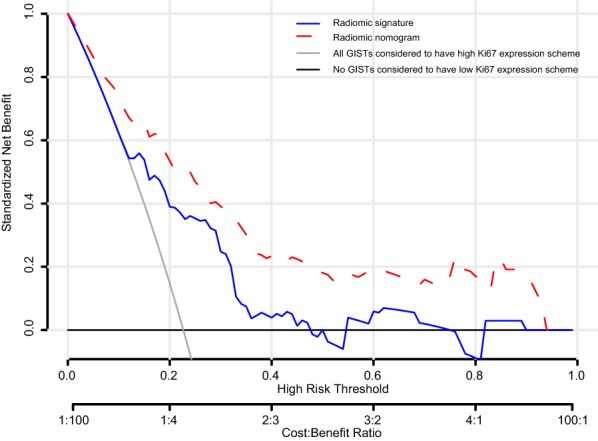
Decision curve analysis for the radiomic signature and radiomic nomogram in prediction of high Ki67 expression in patients with gastrointestinal stromal tumors

In this study, benefits of the radiomic signature and radiomic nomogram were compared with all patients considered to have high Ki67 expression pattern and no patients considered to have high Ki67 expression profile by using the decision curve. Based on the result, we speculate that patients would benefit more from the radiomic signature and the radiomic nomogram if the threshold probability in the clinical decision was set at < 40% (Fig. 6).

## Discussion

This study is the first-ever study, where we have developed and validated an effectively preoperative radiomic nomogram using CT images to predict the Ki-67 expression status in GISTs patients. In this study, radiomic signature and tumor size was included in the nomogram, to identify GISTs with high Ki-67 expression using a relatively large database from four different hospitals. The constructed nomogram provided a noninvasive, preoperative tool to assess Ki-67 expression in GISTs. Our results suggest that this radiomic nomogram can potentially be used to identify the Ki-67 expression status in the etiology of GIST.

Radiomics can extract hundreds of quantitative features from medical images and is promising in prediction the biological behavior on the onset of tumor. In a number of previous studies, radiomics has been implicated in the predictions of the biological behaviors in GISTs [19–21]. Two studies used radiomic features extracted from CE-CT to build prediction models for predicting malignant potential with promising accuracy [19, 20]. Moreover, one study also built a prediction model for mitotic count with AUC of 0.820, 0.769 in the training cohort and the validation cohort, respectively [19]. This suggests that radiomic features may predict Ki-67 expression status in the etiology of GISTs because of positive association of Ki-67 expression and malignant potential [7–12].

Association between radiomic features and Ki-67 expression in various tumors is well documented [32–38]. Most studies focused on MRI texture features to predict the Ki-67 expression in thyroid cancer [37], breast cancer [36], liver cancer [34], and glioma [35] with AUC higher than 0.75. It is noteworthy that only two studies have focused on the CE-CT texture features for the prediction of Ki-67 expression in lung cancer [32, 38]. Results from the above preliminary studies indicated feasibility of radiomic signatures in prediction of Ki-67 expression but none of studies have validated the feasibility and application of radiomic signatures in external validation datasets. In addition, no study has ever investigated whether it is feasible to predict the expression level of Ki-67 based on radiomic signature. To the best of our knowledge, the present study is the first study to develop a radiomic nomogram in the prediction of high Ki-67 expression. In addition, our study also validated predictive efficacy of the radiomic nomogram in the internal validation cohort and external validation cohort from another 3 hospitals with similar results to that of the training cohort.

Ki-67 is expressed in the proliferating cells in the G1, S and G2 phases of the cell cycle, and is a suitable marker to predict the proliferating status compared to the mitotic count, representing proliferating cells during the M stage [6, 7]. Previously, Ki-67 expression levels have been implicated in the clinical set up in predicting malignant potential of GIST [11, 39–41]. In addition, Ki-67 expression could also precisely sub-divide high-risk GISTs effectively with different outcomes and GISTs with high Ki-67 expression also showed poorer prognosis even with imatinib therapy [42]. As an effective complementation of modified NIH criteria, preoperative assessment of Ki-67 expression could provide additional information for clinical decision-making. In the present study, the radiomic nomogram incorporated tumor size and radiomic signature consisted of six radiomic features and had a AUC of more than 0.75 for predicting the high Ki-67 expression in the three independent datasets. This observation indicated the feasibility of preoperative, noninvasive assessment of Ki-67 in GISTs. In addition, developed nomogram in this study potentially can predict the high Ki-67 expression with PPV more than 85% in the validation cohort. About 70% of accuracy could be achieved in the three independent cohorts. With application of our built radiomic nomogram for prediction of Ki-67 expression and radiomic models for prediction of malignant potential or mitotic count [19, 32], comprehensive preoperative assessment could be performed for sequent choice of treatment, including follow-up, endoscopic resection or surgery.

Our study accounts for a number of limitations that we would like to highlight.. All data were collected retrospectively and thus bias could not be avoided. However, subsequent patients were enrolled to reduce selection bias in this study. Further prospective study is needed to validate our radiomic signature and the radiomic model. In this study, CT parameter varied among participating hospitals, leading to data heterogeneity. To address this discrepancy, all selected CT slices were normalized and resampled to adjust for bias by different CT parameters setting in different hospitals before extraction of all radiomic features. In addition, we also applied z-score method to standardize the radiomic features using mean and standard deviation calculated from the training cohort. In our study, AUC also did not differ significantly among different hospitals, which indicated reliable normalization method. In our study, gene mutations were not included for development of the predictive model of high-malignant potential GISTs in the present study. However, these variables could not be obtained by preoperative clinical examination and thus were not taken into consideration. Further study is required to investigate the relationship between radiomic features and gene mutation.

## Conclusion

In conclusion, a radiomic nomogram on the basis of radiomic signature from CE-CT and tumor size was built for prediction of Ki-67 expression in GISTs. The proposed nomogram provides an ideal model and reference for the future preoperative assessment of cell proliferation on the onset of GISTs. However, we must attest that even though our data are promising, being the very study of its kind, these results are preliminary and further studies are required to validate our findings, primarily to assess the potential for clinical translation.

## Supplementary information

**Additional file 1: A1.** CT examinations; **A2.** Radiomic features extraction; **A3.** Radiomic feature selection and signature building process; **A4.** Radiomics signatures calculation formula; **Table S1.** The CT protocol of the four centers.

## Data Availability

The datasets generated and/or analysed during the current study are not publicly available due personal information involved but are available from the corresponding author on reasonable request.

## Abbreviations

CE-CT: Contrast-enhanced computed tomography
GIST: Gastrointestinal stromal tumor
LASSO: Least absolute shrinkage and selection operator
CI: Confidence interval
NIH: National Institutes of Health
NCCN: National Comprehensive Cancer Network
AFIP: Armed Forces Institute of Pathology
ROI: Region of interest
GLCM: Grey-level co-occurrence matrix
GLRLM: Grey-level run-length matrix
GLSZM: Grey-level size-zone matrix
GLDM: Gray-level dependence matrix
ICC: Inter-class correlation coefficients
ROC: Receiver operating characteristic curve
AUC: Area under receiver operating characteristic curve
MI: Mutual information
mRMR: Minimum redundancy maximum relevance
