# Supplementary material for: Personalized CT-based radiomics nomogram preoperative predicting Ki-67 expression in gastrointestinal stromal tumors: a multicenter development and validation cohort
Source: Clin Transl Med. 2020 Jan 31;9:12. doi: 10.1186/s40169-020-0263-4 (PMC6994569; doi:10.1186/s40169-020-0263-4)
Supplement: Supplementary file 1 — Additional file 1: A1. CT examinations; A2. Radiomic features extraction; A3. Radiomic feature selection and signature building process; A4. Radiomics signatures calculation formula; Table S1. The CT protocol of the four centers. [file 40169_2020_263_MOESM1_ESM.docx]

**Additional file A1: CT examinations**

All patients in the four centers underwent contrast-enhanced abdominal CT in similar scan setup but with different systems and parameters. For the stomach scan, all patients were recommended an overnight fast, and 20 mg of scopolamine or anisodamine were administered intramuscularly to reduce gastrointestinal peristalsis 15–20 min prior to computed tomography (CT) examination. For the small bowel scan, all patients were recommended an overnight fast, and then administered with either 2L or 3L polyethylene glycol (PEG) before the day of examination or 1L to 2L mannitol on the day of examination. For large bowel scan, all patients were recommended to stay empty stomach for atlast 4 to 6 hours on the day of examination. Patients were advised to consume 600ml to1000 ml warm water or received 6 gm of effervescent granules to distend the stomach prior to the CT examination.

The CT scans, covering the entire stomach region or bowel region, were acquired during a steady breath condition by positioning the patient supine. The CT image acquisition parameters of the four centers were shown in Table S1. The diagnosis was performed with a standard dynamic window adjustment procedure, on window-adjustable PACS work stations. A narrow window was used to demonstrate the primary GIST. Three-plane images (axial, coronal, and sagittal) were observed together to facilitate the detection and location of the primary GIST.

**Additional file A2: Radiomic features extraction.**

Before extraction of radiomic features, all the arterial phase CT images were subjected to image normalization (the intensity of the image was scaled to 0–500) and resampled to the same resolution (1mm×1mm×1mm) before the feature extraction to avoid any possible data heterogeneity. This procedure was followed by a filtering process to implement image smoothing and image difference before the CT radiomic feature extraction were executed. Separate filters were used to avoid the multi-dimensional convolution. The convolution was performed with a low-/high-pass “Coiflet 1” wavelet filter along the x-/y-direction by applying different weights to band-pass and sub-bands (LHL, LHH, LLH, HLL, HHL and HLH) of the tumor region as compared to low frequency and high frequency sub-bands (LLL and HHH) in the wavelet domain. Following the filtering, a total of 833 quantitative features were extracted from the ROI of the original image and its corresponding filtered results, including 18 features of first order statistics, 14 features of shape, 22 features of grey-level co-occurrence matrix (GLCM), 16 features of grey-level run-length matrix (GLRLM), 16 features of grey-level size- zone matrix (GLSZM), 14 gray level dependence matrix GLDM), 5 features of gray-level dependence matrix (GLDM) and 728 features of wavelet features. The process of image filtering and feature extraction was performed using PyRadiomics on Python (3.7) [1].

Radiomic features of all patients were standardized by using the z-score method, which is based on the parameters calculated from the training cohort.

**Additional file A3: Radiomic feature selection and signature building process**

One radiologist with 9 years of experience with CT (reader 1) performed segmentations for all patients. Three months after the initial segmentation, 40 patients in the training cohort were randomly selected and re-segmented by the same radiologist to assess intra-reader agreement in radiomic features. This procedure was followe by a segmentation process executed by another radiologist with 5 years of experience with CT (reader 2) for inter-reader agreement assessment.

The feature selection and signature building process were performed for three steps. First step, based on the different independent segmentation groups, intra-/inter-class correlation coefficients (ICCs) were used to estimate each feature’s reproducibility [2]. Stable features with ICCs >0.8 were reserved. Features were then ranked using the minimum redundancy maximum relevance (mRMR) algorithm [3] by calculating the mutual information (MI) between radiomics features and risk classification of GISTs and only 30 highest-ranking features in mRMR were reserved.

For radiomic signature building, the Least Absolute Shrinkage and Selection Operator Method (LASSO) logistic regression model was used to build Radiomics signature [4].

**Additional file A4: Radiomics signatures calculation formula**

Radiomics score= -0.843 + 0.035 × wavelet.HHH_glszm_LargeAreaLowGrayLevelEmphasis – 0.019 × wavelet.HLL_glcm_MaximumProbability + 0.0002 × wavelet.HHH_glcm_Imc2 + 0.061 × wavelet.LLL_ngtdm_Busyness - 0.010 × wavelet.HLL_gldm_LargeDependenceEmphasis + 0.287 × wavelet.LLL_firstorder_TotalEnergy

**Table S1.** The CT protocol of the four centers

| Parameters | Center 1 | Center 2 | Center 3 | Center 4 |
| --- | --- | --- | --- | --- |
| CT version | Spectral CT (Discovery CT750 HD scanner, GE Healthcare, USA) | Spectral CT (Aquilion One TSX-301A, TOSHIBA, Japan) | Spectral CT (SOMATOM Sensation 16,  Siemens Forchheim Germany; Siemens Definition AS 40,  Siemens Healthcare Forchheim Germany; SOMATOM  Definition Flash, Siemens Healthcare Forchheim Germany;  LightSpeed VCT, GE Healthcare, Milwaukee, WI) | Spectral CT (Discovery CT750 HD scanner, GE Healthcare, USA; Lightspeed VCT scanner, GE Healthcare, USA;  BrightSpeed, GE Healthcare, USA) |
| CT  tube voltage | 120 kVp | 120 kVp | 120 kVp | Spectral imaging mode switching between 120 kVp and 140 kVp |
| CT  tube current | 220 mA | 60 mA | 300 mA or 100-300 mA | 50-480mA (the duration of automatically optimized to provide similar signal strength) |
| CT  rotation time | 0.6 s | 0.6 s | 0.5 s or 0.6 s | 0.60-0.80 s |
| CT detector collimation | 64×0.625 mm | 64×0.5 mm | 16×1.2 mm or 32×1.2 mm or 64×0.625 mm | 64×0.625 mm |
| Contrast agent type | Iopamidol, Iopamiro, Bracco Sine, Shanghai, China | Iopamiro, Bracco Sine, Shanghai, China | Iopamidol, Iopamiro, Bracco Sine, Shanghai, China; iohexol, Omnipaque 300, Amersham,  Shanghai, China | Omnipaque, GE Healthcare, USA |
| Contrast agent concentration | 370 mgI/ml | 300 mgI/ml | 300 mgI/ml | 300 mgI/ml |
| Contrast agent dosage | infused 1.5 ml/kg body weight | infused 1.5 ml/kg body weight | infused 1.5 ml/kg body weight | infused 1.5 ml/kg body weight |
| Contrast agent infused rate | 3.0 ml/s | 3.0 ml/s | 3-4 ml/s | 2.0~3.3ml/s |
| Arterial phase  interval time | 35 s after injection of  contrast agent | 36 s after injection of  contrast agent | 30 s after injection of  contrast agent | 30 s after injection of  contrast agent |
| Venous phase  interval time | 70 s after injection of  contrast agent | 90 s after injection of  contrast agent | 60 s after injection of  contrast agent | 65~70 s after injection of  contrast agent |
| Image matrix | 512×512 | 512×512 | 512×512 | 512×512 |
| Field of view | 500×500 mm | 500×500 mm | 500×500 mm | 500×500 mm |
| Reconstruction image thickness | 2 mm or 5 mm | 5 mm | 1.2 mm or 2 mm or 5 mm | 5 mm |

**Reference**

[1] van Griethuysen JJM, Fedorov A, Parmar C, Hosny A, Aucoin N, Narayan V, et al. Computational Radiomics System to Decode the Radiographic Phenotype. Cancer Res. 2017;77:e104-e7.

[2] Shrout PE, Fleiss JL. Intraclass correlations: uses in assessing rater reliability. Psychological bulletin. 1979;86:420.

[3] De Jay N, Papillon-Cavanagh S, Olsen C, El-Hachem N, Bontempi G, Haibe-Kains B. mRMRe: an R package for parallelized mRMR ensemble feature selection. Bioinformatics (Oxford, England). 2013;29:2365-8.

[4] Friedman J, Hastie T, Tibshirani R. Regularization paths for generalized linear models via coordinate descent. Journal of statistical software. 2010;33:1.
